# Exploiting Ubiquitination: African Swine Fever Virus-Mediated Recruitment of Host E3 Ligases During Viral Infection and Immune Regulation

**DOI:** 10.3390/pathogens15070716

**Published:** 2026-07-07

**Authors:** Kiramage Chathuranga, W. A. Gayan Chathuranga, Tania F. de Koning-Ward, Jong-Soo Lee

**Affiliations:** 1 School of Medicine, Deakin University, Geelong 3216, Australia; tania.dekoning-ward@deakin.edu.au; 2 Institute for Mental and Physical Health and Clinical Translation (IMPACT), Deakin University, Geelong 3216, Australia; 3 Department of Microbiology and Immunology, College of Medicine, University of Illinois, Chicago, IL 60612, USA; gayanc@uic.edu; 4 College of Veterinary Medicine, Chungnam National University, Daejeon 34134, Republic of Korea; jongsool@cnu.ac.kr

**Keywords:** African swine fever virus, E3 ligase, post-translational modification

## Abstract

Ubiquitination is a post-translational modification that governs various facets of eukaryotic biology, including protein stability, signaling, and immune regulation. The modification process is mediated by a coordinated enzymatic cascade, in which E3 ubiquitin ligases confer substrate specificity and determine the functional outcome of ubiquitin attachment. In the case of a virus infection, host cellular signaling networks undergo major ubiquitin-dependent changes to protect the host cell, including remodeling of cellular organelles, coordination of innate immunity, and reprogramming of metabolic pathways to prevent virus replication. African swine fever virus (ASFV) has evolved numerous strategies to counteract or evade these responses, thereby manipulating host defenses and promoting its replication. By modulating ubiquitination-dependent host cellular functions, the virus can regulate key immune signaling factors, suppress interferon production, and interfere with inflammatory pathways. These actions not only antagonize antiviral defenses but also remodel cellular homeostasis to favor infection. The important interplay between host defense and viral manipulation underscores the versatility of the ubiquitin system as a battleground in ASFV infection. In this review, we discussed mechanistic insights into how ASFV subverts ubiquitin pathways during host–virus interactions. This comprehensive knowledge might be beneficial for pharmaceutical exploration of host E3 ligase-dependent anti-ASFV treatment.

## 1. Introduction

African swine fever virus (ASFV) is the causative agent of African swine fever (ASF), a lethal and economically devastating disease of domestic pigs and wild boar. The first recorded ASFV outbreak occurred in Kenya in 1921 [1]. For many years, ASFV was confined to the African continent, but its geographical range shifted in 2018 to Asia. Since then, ASFV has spread to more than 40 countries worldwide [2]. The first outbreak in China was confirmed in August 2018, and the virus spread at an exceptional pace. Within less than a year, it reached every province and autonomous region of the country. It is estimated that 300 million pigs were lost through either death or compulsory culling, and the resulting economic burden was valued at more than 100 billion U.S. dollars [3]. ASFV circulates naturally in East Africa through a sylvatic cycle involving warthogs and soft ticks, such as *Ornithodoros* ticks, with infections typically subclinical or asymptomatic. In contrast, outbreaks in domestic pigs and wild boar often result in acute hemorrhagic fever with mortality up to 100%. Some isolates of lower virulence have been described, producing chronic infections, yet these remain a significant challenge for control [3].

ASFV is a large arbovirus belonging to the family *Asfarviridae*. It is a double-stranded DNA virus with a 170–190 kb genome that comprises 150–200 open reading frames (ORFs) encoding 68 structural proteins and more than 100 nonstructural proteins [4]. However, the functions of most of these proteins remain largely unclear. The limited knowledge of defined protective antigens, the unclear correlates of protective immunity, and the ability of the virus to evade host immunity have all hindered effective vaccine development. Moreover, given its persistence in wildlife reservoirs, ASFV will be difficult to eradicate. Although intensive research has been carried out worldwide since the major epidemic of 2018, a World Organization for Animal Health (WOAH) accepted vaccine that is both safe and broadly effective has yet to be developed. In 2023, Vietnam became the first country to authorize the use of a modified live vaccine, but several studies have highlighted issues related to these vaccines’ safety, genetic stability, virulence, and efficacy under different field conditions [5,6,7,8].

It is not surprising that during infection, pathogens must overcome host defenses and manipulate cellular mechanisms to establish efficient, persistent infection and to initiate viral pathogenesis. From the perspective of pathogens, evolutionary adaptation has enabled them to exploit numerous strategies to subvert host defense mechanisms and enhance their replication. Among them, the ubiquitin-proteasome system (UPS) is one of the main targets of many pathogens [9]. Host E3 ubiquitin ligases, which confer substrate specificity within UPS, are often targeted by pathogens to remodel the intracellular environment in their favor. By recruiting or hijacking host E3 ligases, pathogen proteins can direct the ubiquitination or prevent ubiquitination of key cellular factors, thereby modulating signaling pathways, metabolic processes, and innate immune responses to create a cellular state that is favorable for their replication [10]. For example, disruption of normal ubiquitination events can affect protein turnover, intracellular trafficking, and signal transduction, all of which are critical for establishing infection [11].

Ubiquitin is a small, highly conserved protein (8 kDa) present in all eukaryotic cells and is covalently conjugated to target proteins as a post-translational modification. This process, known as ubiquitination, is carried out through a three-enzyme cascade involving ubiquitin-activating (E1), ubiquitin-conjugating (E2), and ubiquitin-ligase (E3) enzymes. Among these, E3 ligases confer substrate specificity by recognizing target proteins and facilitating the transfer of ubiquitin from the E2 enzyme to specific lysine residues on the substrate [12]. E3 ligases form a structurally and functionally diverse superfamily, broadly divided into four classes: Homologous to E6AP C-terminus (HECT), Really Interesting New Gene (RING) finger, RING-in-between-RING (RBR), and U-box types. HECT ligases form covalent intermediates with ubiquitin before transferring it to substrates, whereas RING ligases act as scaffolds that bring E2 and substrates into proximity for direct transfer [12]. RBR ligases combine features of both systems, while U-box ligases resemble RING ligases but lack a zinc-finger domain [13]. This diversity equips ubiquitination with the versatility to regulate numerous cellular pathways. A single ligase can act on multiple substrates, and precise control of E3 ligase expression, activity, and stability is essential to prevent aberrant ubiquitination or auto-ubiquitination [14]. Regulation of E3 ligases occurs through tissue-specific expression, temporal cues, environmental signals, and substrate availability, ensuring that protein ubiquitination is tightly coordinated [15].

Understanding the mechanisms by which pathogens exploit host E3 ligases provides insight into the molecular strategies underpinning pathogen replication and pathogenesis. Such knowledge is essential for the development of therapeutics aimed at restoring normal ubiquitin-mediated regulation and reinforcing host cellular defenses. Here, we review recent research on the recruitment of host E3 ligases by ASFV proteins and discuss contributions that have increased our understanding of host–pathogen interaction.

## 2. Protein Ubiquitination in Eukaryotes

Ubiquitination is carried out by the enzymatic cascade. Firstly, ubiquitin is activated in an ATP-dependent reaction, leading to the formation of a thioester bond between the C-terminal glycine of ubiquitin and a cysteine residue on the E1 enzyme. The activated ubiquitin is then transferred to the active cysteine of an E2 enzyme. Subsequently, the E3 ligase bridges the E2 and the substrate, guiding the transfer of ubiquitin either directly or indirectly onto a lysine residue of the target protein through the formation of an isopeptide bond [16]. This last step is the key determinant of substrate specificity and is responsible for the functional diversity of the ubiquitin system. This versatility arises from several critical modification sites, including seven lysine residues (K6, K11, K27, K29, K33, K48, and K63) and the N-terminal methionine (M1). Each of these positions in ubiquitin can serve as a linkage point for the assembly of ubiquitin chains. Among these, K48 and K63-linked polymers are the most extensively studied in eukaryotic cells [16,17]. K48-linked chains typically mark proteins for recognition and degradation by the 26S proteasome, whereas K63-linked chains participate largely in non-proteolytic roles, such as DNA repair, signal transduction, and autophagy [18]. Other chain types, although less well characterized, are increasingly recognized as regulators of distinct pathways. K6-linked chains have been implicated in DNA damage responses, spermiogenesis, and metabolic diseases [19,20], K11-linked chains in cell cycle control and protein degradation [21,22,23] and K27-linked chains in processes ranging from protein secretion, protein degradation, and mitochondrial quality control [24].

Beyond chain diversity, ubiquitination can occur in several distinct forms. Mono-ubiquitination refers to the attachment of a single ubiquitin to one lysine residue on a substrate, often regulating activity or localization without triggering degradation [25,26]. Multi-mono-ubiquitination involves a single ubiquitin attached to several different lysines on the same substrate [27,28]. Polyubiquitination, by contrast, generates extended chains from one lysine residue, with outcomes determined by the linkage type [29]. Together, these different modes of ubiquitination provide an important system for fine-tuned control of protein fate and function.

## 3. Targeting the Host Ubiquitination System in ASFV Infections

Accumulating evidence has demonstrated that viruses can regulate host proteins by recruiting host E3 ubiquitin ligases to manipulate host immune response or promote virus replication. For example, Newcastle disease virus (NDV) V protein degrades mitochondrial antiviral signaling protein (MAVS) by recruiting the E3 ubiquitin ligase RNF5 to polyubiquitinate MAVS [30], while pseudorabies virus (PRV) tegument protein UL13 recruits RNF5 to inhibit Stimulator of Interferon Genes (STING)-mediated antiviral immunity [31]. Many ASFV proteins play critical roles in suppressing host defenses to facilitate replication and persistence [32]. Among them, recent research has focused on identifying host E3 ubiquitin ligases that are hijacked by ASFV proteins during infection to facilitate virus replication. As a control measure, there has been significant interest in creating inhibitors targeting viral proteins [33,34]. However, the capacity of viruses to evolve rapidly and develop resistance to inhibitors remains a significant obstacle. This has highlighted the importance of identifying host factors that are essential for viral replication. Targeting such host proteins offers a promising strategy for therapeutic intervention, as it may provide more durable control options against this devastating virus.

Indeed, one important strategy employed by ASFV to promote virulence and survival is interaction with host E3 ubiquitin ligases, most often to drive the degradation of key host factors, thereby enhancing viral replication. In the following sections, the ASFV proteins that interact with host E3 ligases from several essential signaling pathways are described. To our knowledge, this is the first review to focus on ASFV proteins that target host E3 ubiquitin ligases and to provide a detailed discussion of the underlying molecular mechanism.

## 4. ASFV Proteins Disrupt Type I Interferon Signaling by Recruiting Host E3 Ubiquitin Ligases

Type I interferons (IFNs), pro-inflammatory cytokines, and antiviral proteins play an important role in a host’s defense against invading viruses. To initiate the signaling, the sensor cyclic GMP-AMP (2′3′cGAMP) synthase (cGAS) is primarily responsible for recognizing cytosolic viral DNA, while Retinoic acid-inducible gene I (RIG-I) and Melanoma Differentiation-Associated protein (MDA)-5 bind to viral RNA in the cytoplasm. cGAS undergoes a reconfiguration of its catalytic pocket before binding its substrates, adenosine triphosphate (ATP) and guanosine triphosphate (GTP), which are then used to generate the second messenger 2′3′cGAMP [35]. The synthesized 2′3′cGAMP molecule attaches itself to the endoplasmic reticulum (ER) membrane adaptor STING, thereby enhancing the structural modifications that are required for its activation. Once activated, STING relocates to the ER Golgi intermediate compartment, where it recruits and activates TANK-binding kinase 1 (TBK1) [36]. On the other hand, RIG-I and MDA5 interact with the downstream adaptor molecule MAVS. This interaction causes aggregation of MAVS to form a prion-like protein complex, which relays the signal to kinases such as TBK-1 [37]. TBK1 then triggers the phosphorylation of interferon regulatory factor (IRF)-3 [38]. Activation of this cascade results in phosphorylation of the transcription factors IRF-3 and IRF-7. Finally, nuclear translocation of IRF-3 and IRF-7 induces the expression of type I IFN genes [37].

**Figure 1:**
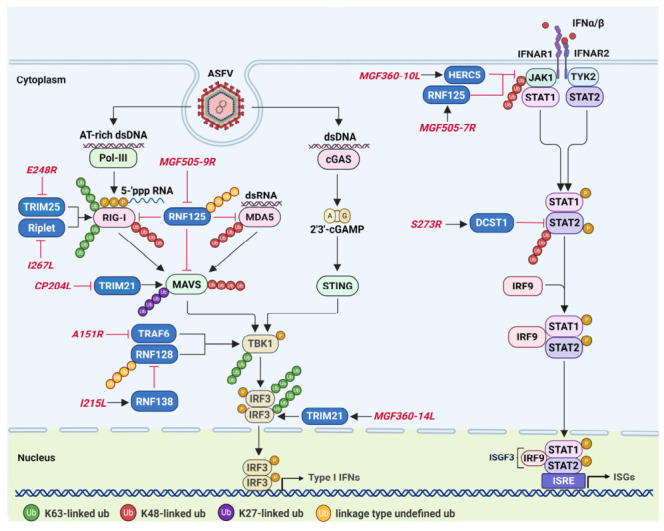
Overview of ASFV proteins that hijack or interfere with host E3 ubiquitin ligases and manipulate the type I IFN signaling pathway during virus infection. The diagram summarizes the type I IFN signaling pathway targeted by ASFV proteins through direct modulation of ubiquitin-dependent signaling. Host factors, including E3 ligases, adaptor proteins, and signaling intermediates, are indicated at their respective subcellular locations. Ubiquitin linkages are color-coded as K63-linked (green), K48-linked (red), K27-linked (purple), or undefined (yellow). (ub: Ubiquitination).

Secreted type I IFN then binds to the IFN receptors on the cell surface. This receptor is composed of two subunits, IFNR1 and IFNR2, which are associated with Janus kinase (JAK) 1 and JAK2, respectively [39]. Activation of the JAKs results in tyrosine phosphorylation of signal transducer and activator of transcription (STAT) 2 and STAT1. This leads to the formation of STAT1-STAT2- IFN-regulatory factor 9 (IRF9) complexes, also known as IFN-stimulated gene factor 3 (ISGF3) complexes. These complexes translocate to the nucleus and bind IFN-stimulated response elements (ISREs) in DNA to initiate gene transcription [40,41]. Previous studies suggest that diverse ASFV proteins play multiple roles in blocking type I IFN signaling, thereby facilitating innate immune evasion and enabling successful replication of ASFV within macrophages [32]. Below, we describe the immunomodulatory mechanisms by which ASFV proteins disrupt type I IFN signaling via recruiting host E3 ubiquitin ligases (Figure 1, Table 1 and Table 2).

### 4.1. ASFV I267L Binds with Host E3 Ubiquitin Ligase Riplet

The *I267L* gene of ASFV encodes a 267-amino-acid transmembrane protein that is expressed during the early phase of infection. *I267L* is a highly conserved and unique protein, with 100% amino acid (aa) identity among strains of the p72 genotype I and II ASFV, suggesting evolutionary pressure to maintain its function in the viral life cycle [58,59]. Various in vivo studies have yielded conflicting conclusions as to the functional significance of this protein. In pigs, inoculation with the highly virulent ASFV CN/GS/2018 strain lacking *I267L* caused noticeably elevated IFN-β levels, and the virus has reduced virulence and pathogenicity [42]. In contrast, Zhang et al. demonstrated that a virulent ASFV SY18 strain with the *I267L* gene deletion (SY18Δ*I267L*) did not exhibit altered replication or virulence [59]. The conflicting findings regarding the role of *I267L* in ASFV virulence likely stem from differences in viral strain backgrounds and experimental parameters. While *I267L* deletion in the CN/GS/2018 isolate significantly attenuated the virus in 80 to 90-pound commercial pigs at a dose of 10 HAD_50_, the same deletion in the SY18 isolate failed to alter virulence or replication in 20 kg Landrace pigs at doses of 10^2^ or 10^5^ TCID_50_. These discrepancies suggest that *I267L*’s contribution to pathogenesis is context-dependent, potentially due to variations in titration methods, animal models, strain-dependent differences in host–virus interactions, or genomic redundancy (particularly among MGF360 and MGF505 family proteins), where other viral proteins might compensate for the loss of *I267L* in certain highly virulent isolates.

A study by Ran et al. revealed that ASFV *I267L* engages with the host E3 ubiquitin ligase protein Riplet (also known as RNF135, RIG-I E3 ubiquitin ligase, or RING finger protein 135) [42]. Riplet is a RING-type E3 ligase and was first identified by Oshiumi and colleagues as an essential component of the RNA polymerase III-RIG-I signaling pathway [60,61]. Riplet promotes type I IFN production during the initial stages of antiviral defense by catalyzing K63-linked polyubiquitination of RIG-I [62]. Structurally, Riplet contains an N-terminal RING domain and C-terminal SPRY/PRY motifs, both required for it to function properly [63,64].

Ran et al. further demonstrated that ASFV *I267L* interferes with the catalytic activity of Riplet, disrupting the interaction between Riplet and RIG-I required for downstream signaling activation [42]. Functional mapping localized the inhibitory effect of *I267L* specifically to RIG-I, without affecting signaling mediated by downstream adaptor proteins in the pathway. However, no direct interaction has been observed between *I267L* and RIG-I. Co-immunoprecipitation assays demonstrated that *I267L* binds to the Riplet, but not to RIG-I or to known RIG-I-targeting negative regulators such as CYLD or USP3 [65,66,67]. Further, *I267L* suppressed Riplet-driven K63-linked ubiquitination of RIG-I. As K63-linked ubiquitination is necessary for RIG-I interaction with the adaptor VISA/MAVS, *I267L* expression impaired RIG-I recruitment to MAVS and blocked subsequent signaling events. Collectively, in vitro findings from this study establish *I267L* as a viral effector that disrupts Riplet-mediated ubiquitination of RIG-I, thereby preventing the activation of innate antiviral defenses. Moreover, *I267L*-deficient ASFV induces higher levels of IFN-β and shows reduced replication in both macrophages and pigs, with virulence and pathogenesis attenuated [42].

### 4.2. ASFV E248R Binds with E3 Ubiquitin Ligase TRIM25

The *E248R* gene of ASFV encodes a 248-amino-acid structural protein that is highly conserved across diverse ASFV isolates and essential for viral replication and spread [68]. Bioinformatic and biochemical analyses classify *E248R* as a type II transmembrane glycoprotein localized within the inner viral envelope and functionally analogous to poxviral entry/fusion complex proteins [68,69]. *E248R* plays a central role in mediating membrane fusion during virus entry and facilitating core penetration into the cytoplasm. ASFV particles lacking functional *E248R* are defective in productive entry; they can bind and be internalized by host cells, but fusion between viral and endosomal membranes is impaired, failing to release the viral core into the cytosol [68]. Studies have further confirmed that *E248R* is indispensable for virus replication in swine macrophages, the primary target cells of ASFV. Deletion of *E248R* produced non-infectious particles that were incapable of sustaining infection. *E248R* interacts with other viral envelope proteins to form a fusion-competent complex [68]. Because the protein is strictly required for initiating infection but is not directly involved in immunomodulation, *E248R* has attracted attention as a potential target for rational vaccine design [68,69,70].

*E248R* exerts its inhibitory effect by directly targeting the E3 ubiquitin ligase TRIM25, which catalyzes the K63-linked polyubiquitination of RIG-I [43]. TRIM25 (also known as Tripartite Motif Containing Protein 25, RNF147, or ZNF147) plays pivotal roles in antiviral innate immunity, immune signaling regulation, and host–pathogen interactions [71,72]. It belongs to the tripartite motif (TRIM) family and, like other TRIM proteins, is characterized by its conserved tripartite architecture. It also harbors an N-terminal RING domain that confers E3 ligase activity, two B-box motifs, and a central coiled-coil domain responsible for homo-oligomerization and interactions with other TRIM proteins. TRIM25 is best known as the primary E3 ligase catalyzing K63-linked polyubiquitination of the cytosolic RNA sensor RIG-I. This modification occurs in the CARD (caspase activation and recruitment domain) region of RIG-I and is essential for its conformational activation. Accordingly, TRIM25 serves as a key gatekeeper of RIG-I-dependent antiviral responses [73,74,75].

Zhao et al. demonstrated that ASFV *E248R* acts as a potent antagonist of type I IFN induction. Expression of *E248R* in porcine cells markedly suppressed IFN-β transcription and downstream antiviral gene expression, which correlated with reduced IRF3 phosphorylation and impaired IFN-β protein production. *E248R* directly interacts with the N-terminal CARD region of RIG-I, an essential domain for its activation. This interaction suppressed K63-linked ubiquitination of RIG-I, an essential post-translational modification for its conformational activation and recruitment of MAVS. Consistent with this, E248R impaired the physical interaction between RIG-I and MAVS in a dose-dependent manner, thereby preventing assembly of the signaling platform required for IRF3 phosphorylation and type I IFN production. *E248R* interacts with the coiled-coil domain (CCD) of TRIM25, a region essential for its dimerization and higher-order multimerization. By disrupting CCD-dependent multimerization, *E248R* effectively inhibits the enzymatic activity of TRIM25, thereby preventing the conjugation of K63-linked ubiquitin chains onto RIG-I. This interference not only reduces RIG-I ubiquitination but also weakens the subsequent interaction between TRIM25 and RIG-I, further downregulating RIG-I activation. Furthermore, loss of *E248R* enhances ASFV-mediated phosphorylation of IRF3 and IFN-β protein levels [43].

### 4.3. ASFV MGF505-9R Binds with Host E3 Ubiquitin Ligase RNF125

The *MGF505-9R* gene of ASFV encodes a nonstructural, early-stage protein that belongs to the multigene family 505 (MGF505), which typically consists of 8 to 11 members located near the ends of the viral genome. *MGF505-9R* functions as an important protein in immune regulation during infection [44,76].

A study by Zhao et al. revealed that the ASFV non-structural protein *MGF505-9R* interacts with the host E3 ubiquitin ligase RING finger protein 125 (RNF125) (also known as TRAC-1, T cell RING finger protein) [44]. RNF125 is a RING-type E3 ubiquitin ligase that plays key roles in regulating immune signaling and antiviral responses. By degrading RIG-I, p53, and related adaptors, RNF125 attenuates antiviral signaling and modulates the amplitude of IFN responses, preventing excessive inflammation and maintaining immune homeostasis [77,78]. Zhao et al. further demonstrated that *MGF505-9R* enhances RLR signaling by promoting the autoubiquitination and degradation of RNF125. Mechanistically, *MGF505-9R* directly binds to RNF125, triggering the host ligase’s intrinsic autoubiquitination activity and leading to its depletion. Functional mapping and co-immunoprecipitation assays showed that *MGF505-9R* dose-dependently inhibits the association between RNF125 and its substrates (RIG-I, MDA5, and MAVS), thereby significantly reducing their K48-linked ubiquitination. While *MGF505-9R* increases the protein stability of these upstream RLR components, it does not alter their mRNA levels or affect their K63- or K11-linked ubiquitination status. This stabilization results in hyperactivation of downstream signaling, characterized by increased phosphorylation of IRF3 and NF-κB, which ultimately promotes the expression of IFN-β and IL-1β. Moreover, infection with an *MGF505-9R*-deficient ASFV strain induces significantly lower levels of IFN-α, IFN-β, and IL-1β in porcine alveolar macrophages compared to the parental virus. Collectively, the findings of this study establish *MGF505-9R* as a novel viral effector that positively regulates the host’s innate immune response by antagonizing a negative regulatory factor. This mechanism provides a molecular explanation for the severe inflammatory responses frequently observed during ASFV infection [44].

### 4.4. ASFV CP204L Binds with E3 Ubiquitin Ligase TRIM21

The *CP204L* gene encodes a highly antigenic phosphoprotein, commonly known as p30 (or p32), that is approximately 201 aa long and is synthesized during the early phase of ASFV infection [45,79]. *CP204L* is highly conserved across all known ASFV isolates and is among the most abundant viral proteins. Subcellular localization studies indicate that *CP204L* is predominantly found in the cytoplasm, but it is also associated with the nucleus, the plasma membrane, and the periphery of cytoplasmic virus factories [79,80]. By facilitating host–cell attachment and internalization, *CP204L* is a multifunctional gene indispensable for viral replication [81]. *CP204L* has been reported to restrict antiviral type I IFN induction by targeting host TRIM21 and the signaling adaptor MAVS, thus evading host innate immune defenses [45]. *CP204L* interacts with the host HOPS complex component VPS39, disrupting normal endolysosomal trafficking and promoting lysosome clustering around viral replication sites, thereby facilitating the formation and maintenance of virus factories [79]. Moreover, the inhibition of the *CP204L* gene, such as through CRISPR/Cas9 technology, significantly reduces virus yields and spread, making it a critical target for vaccine development and serological detection [81].

A study by Zhang et al. revealed that the ASFV early structural protein *CP204L* interacts with the host E3 ubiquitin ligase TRIM21 and the signaling adaptor MAVS to facilitate immune evasion [45]. TRIM21 (also known as Tripartite Motif-Containing Protein 21, RING Finger Protein 81, or Ro52) is a multifunctional E3 ubiquitin ligase with critical roles in innate immunity. TRIM21 functions as a positive regulator of the RIG-I-like receptor (RLR) pathway by catalyzing the K27-linked polyubiquitination of MAVS, which is essential for the downstream induction of type I IFNs [82]. The study found that during ASFV infection, *CP204L* directly interacts with both TRIM21 and MAVS, forming a tripartite *CP204L*-TRIM21-MAVS complex in the cytoplasm. Zhang et al. further demonstrated that *CP204L* suppresses activation of antiviral signaling by counteracting TRIM21-mediated K27 polyubiquitination of MAVS. Mechanistically, this inhibition prevents the recruitment of TBK1 to the MAVS complex, thereby blocking the downstream phosphorylation of TBK1 and IRF3 required for IFN induction. Domain mapping indicated that the C-terminal region of *CP204L* is critical for these interactions, specifically binding to the PRY/SPRY domain of TRIM21 and the PRO/C-terminal domains of MAVS. Functional assays showed that while ectopic expression of TRIM21 inhibits ASFV replication, the presence of *CP204L* effectively reverses this inhibitory effect by dampening the RLR-mediated response. Collectively, findings of this study establish *CP204L* as a key viral effector that disrupts host innate immunity by targeting the post-translational modification of the central RLR adaptor, MAVS [45].

### 4.5. ASFV A151R Binds with E3 Ubiquitin Ligase TRAF6

The *A151R* gene encodes a 151-amino-acid, non-structural 17 kDa protein that is expressed during the early phase of ASFV infection. *A151R* is highly conserved across diverse ASFV isolates [83]. Subcellular localization studies indicate that *A151R* predominantly associates with host mitochondria, where it contributes to regulating cellular redox homeostasis and mitochondrial integrity during infection. By stabilizing mitochondrial homeostasis and modulating cell survival pathways, *A151R* supports viral persistence and limits premature immune clearance [46,84]. *A151R* has been reported to affect the function of TBK1 and IκB kinase (IKK)ε, thus restricting IFN-β induction and ASFV evasion [46]. Moreover, the recombinant virus ASFV-∆*A151R* has a reduced replication rate and virulence in domestic pigs, and prior inoculation has given protection against challenge with the virulent parental virus [83].

Recent work by Li et al. has characterized ASFV *A151R* as a potent viral antagonist of the cGAS-STING and TBK1 signaling axis, a central pathway driving type I IFN production [46]. Specifically, *A151R* targets the host E3 ubiquitin ligase TRAF6 (also known as TNF receptor-associated factor 6 or RING Finger Protein 85), an adaptor protein and E3 ubiquitin ligase that plays a pivotal role in innate immunity and inflammation. Structurally, TRAF6 is characterized by an N-terminal RING finger domain that confers E3 ligase activity, followed by multiple zinc finger motifs, a coiled-coil region that facilitates homo and hetero-oligomerization, and a C-terminal TRAF domain that mediates receptor and adaptor interactions [85,86]. TRAF6-mediated ubiquitination promotes TBK1 phosphorylation, dimerization, and subsequent activation of IRF3 and NF-κB [87]. Therefore, TRAF6 has been a frequent target of various viral proteins for the manipulation of innate immune activation. Such viral proteins include the avibirnavirus VP3 protein, the influenza D virus Matrix protein 1, and the NDV-M protein [88,89,90].

In HEK 293T cells expressing *A151R*, TBK1 protein expression and phosphorylation were impaired following cGAS-STING stimulation. *A151R* physically interacts with TRAF6, colocalizing at perinuclear compartments and disrupting TRAF6-TBK1 complex formation. Ubiquitination assays further demonstrated that *A151R* expression decreased K63-linked polyubiquitination of TBK1. The apoptotic inhibitor Z-VAD-FMK inhibited *A151R*-mediated TRAF6 degradation. *A151R* contains a histidine on 102 and cysteines on 109, 132, and 135 sites that could coordinate a Zn^2+^ ion to form a Zn-binding motif [84]. Substitution mutations at these sites abrogated the ability of *A151R* to degrade TRAF6, suppress TBK1 phosphorylation, and inhibit IFN-β induction, while preserving its interaction with TRAF6 [46].

### 4.6. ASFV I215L Binds with E3 Ubiquitin Ligase RNF138

The *I215L* gene encodes a 212-amino-acid, 24 kDa protein that is expressed during the early phase of ASFV infection (4 h post-infection) [91]. The protein localizes both to viral factories and more broadly within the nucleus and cytoplasm of infected cells [92]. Sequence analysis shows that *I215L* shares significant homology with ubiquitin-conjugating enzymes and is the only known virus-encoded ubiquitin-conjugating enzyme. The cysteine 85 (C85) residue is essential for its enzymatic activity [91]. Silencing of this gene using siRNA has been shown to reduce ASFV replication significantly [91]. Beyond its role in viral infection, the *I215L* protein serves as a multifunctional regulator. It suppresses host innate immunity by blocking NF-κB activation and downregulating type I IFN signaling [92,93]. Moreover, it engages with the host translation machinery, thereby influencing both viral and cellular protein synthesis [94].

In a study by Huang et al., ASFV *I215L* was identified as a critical viral effector that suppresses type I IFN signaling [47]. The authors demonstrated that *I215L* targets TBK1, a central signaling kinase in DNA and RNA-virus-induced immunity, by inhibiting its K63-linked polyubiquitination, which is required for phosphorylation and activation. However, *I215L* does not physically interact with TBK1. Instead, it interacts with RNF138 (also known as Ring Finger Protein 138 or Nemo-Like Kinase Associated Ring Finger Protein, NARF), which promotes K63-linked ubiquitination of TBK1 [95]. Mechanistic studies demonstrate that *I215L* enhances the interaction between RNF138 and RNF128, resulting in RNF138-dependent ubiquitin-proteasome degradation of RNF128. This degradation diminishes the ability of RNF128 to catalyze K63-linked ubiquitination of TBK1, thereby blocking its activation. Consistently, *I215L* alone, or in combination with RNF138, strongly suppressed TBK1 ubiquitination. Collectively, findings of this study establish *I215L* as a viral effector that recruits RNF138 to degrade RNF128, thereby abolishing K63-linked ubiquitination of TBK1 and preventing downstream IFN signaling [47].

### 4.7. ASFV MGF360-14L Binds with E3 Ubiquitin Ligase TRIM21

The *MGF360-14L* gene of ASFV encodes an approximately 35–38 kDa nonstructural protein that is expressed during the early phase of infection and is conserved among virulent field isolates. Genomic and functional analyses classify *MGF360-14L* as a member of the MGF360, located near the variable termini of the ASFV genome, a region commonly associated with host range and virulence determinants [96]. *MGF360-14L* plays a central role in suppressing the host type I IFN response. *MGF360-14L* inhibits the activation of TBK1 and IRF3 and interacts with DNAJ heat shock protein family (Hsp40) member A3 (DNAJA3), thereby blocking the transcription of IFN-stimulated genes (ISGs) and facilitating efficient virus replication [48,97,98]. Deletion of *MGF360-14L* enhanced the expression of proinflammatory cytokines and apoptotic mediators, suggesting that the gene contributes to immune evasion by maintaining a cellular state favorable for viral persistence [99]. ASFV mutants lacking *MGF360-14L* or neighboring MGF360 genes are attenuated in macrophages and fail to sustain a high level of replication or cause severe disease in pigs, confirming that *MGF360-14L* is a critical virulence factor [99,100].

In the study by Wang et al., *MGF360-14L* was identified as a potent antagonist of type I IFN signaling. The authors demonstrated that it achieves this by recruiting TRIM21 to IRF3, thereby facilitating its K63-linked ubiquitination and degradation [48]. TRIM21 (also known as Tripartite Motif-Containing Protein 21, RING Finger Protein 81, or Ro52) is a multifunctional E3 ubiquitin ligase with critical roles in innate immunity. Expression of *MGF360-14L* in porcine macrophages or PK-15 cells significantly reduced IFN-β transcription and downstream ISG expression following Sendai virus or poly(I:C) stimulation. This suppression correlated with decreased phosphorylation and nuclear translocation of IRF3, indicating that *MGF360-14L* acts upstream of IFN gene activation. Mapping experiments revealed that the C-terminal region of *MGF360-14L* is required for this interaction and for promoting IRF3 degradation. Treatment with the proteasome inhibitor MG132 restored IRF3 levels, confirming the degradation pathway. Mass spectrometry further identified the host TRIM21 as a critical mediator of this process. Knockdown of TRIM21 significantly impaired *MGF360-14L*-induced IRF3 ubiquitination and degradation, confirming that TRIM21 is required for the inhibitory function of *MGF360-14L*. Collectively, these findings demonstrate that *MGF360-14L* suppresses type I IFN responses by targeting IRF3 and hijacking TRIM21 to promote its K63-linked ubiquitination and proteasomal degradation, thereby facilitating ASFV immune evasion [48].

### 4.8. ASFV MGF360-10L Binds with E3 Ubiquitin Ligase HERC5

The *MGF360-10L* gene encodes a 365-amino-acid, 40 kDa protein that is expressed during the early to late phases of ASFV infection [101]. Within infected cells, *MGF360-10L* localizes predominantly in the cytoplasm, particularly near viral replication sites, suggesting a role in orchestrating virus–host interactions during infection [98]. *MGF360-10L* is highly conserved among diverse ASFV isolates [102]. Functional studies using deletion mutants have shown that loss of *MGF360-10L* alone or in combination with other genes (*MGF505-7R*) impairs ASFV replication in primary macrophages and reduces viral virulence in vivo, suggesting that it contributes to proper viral propagation and pathogenesis [101].

Recent work by Li et al. identified ASFV *MGF360-10L* as a viral antagonist of the IFN-β-triggered JAK-STAT pathway through its interaction with JAK1 and the E3 ubiquitin ligase HERC5 (also known as HECT and RLD domain-containing E3 ubiquitin protein ligase 5 or Cyclin E Binding protein 1) [49]. HERC5 is a member of the HECT-type E3 ligase family, characterized by a C-terminal HECT domain and multiple RCC1-like domains that support protein–protein interactions [77,103]. Li et al. demonstrate that overexpression of *MGF360-10L* reduced JAK1 protein abundance without altering its mRNA levels, accompanied by impaired phosphorylation of STAT1 and STAT2 upon IFN-β stimulation. Inhibition experiments confirmed that JAK1 degradation was blocked by the proteasome inhibitor MG132, but not by lysosomal or autophagic inhibitors, underscoring a ubiquitin-proteasome mechanism. *MGF360-10L* expression enhanced the ubiquitination of JAK1, specifically catalyzing K48-linked polyubiquitination, the canonical signal for proteasomal degradation. Mutation of K245 and K269 in JAK1 significantly impaired ubiquitination and stabilized the protein, establishing these sites as critical ubiquitin acceptors for *MGF360-10L*-mediated degradation. Subsequent co-immunoprecipitation experiments confirmed that *MGF360-10L* forms a complex with both HERC5 and JAK1. HERC5 alone can promote JAK1 ubiquitination, while co-expression of *MGF360-10L* and HERC5 further accelerates JAK1 degradation. Moreover, *MGF360-10L*-deficient ASFV induces higher levels of ISGs and shows reduced replication in both macrophages and pigs, with the virulence significantly attenuated in the latter, correlating with lower viral loads in tissues and reduced clinical lesions [49].

### 4.9. ASFV MGF505-7R Binds with E3 Ubiquitin Ligase RNF125

The *MGF505-7R* gene of ASFV encodes a 40 kDa protein that is expressed early during infection and is highly conserved among virulent isolates. As a member of the multigene family 505 (MGF505), *MGF505-7R* localizes to the variable regions of the ASFV genome, a region associated with host range and virulence [78,104]. Functionally, *MGF505-7R* acts as a potent antagonist of the host innate immune response. It suppresses type I IFN production by inhibiting the activation of key signaling molecules, including TBK1 and IRF3, and impairs downstream transcription of ISGs [105,106]. In addition, *MGF505-7R* modulates inflammatory pathways by interfering with NF-κB signaling and preventing inflammasome activation, thereby reducing proinflammatory cytokine release and limiting apoptotic responses [107,108]. Deletion of *MGF505-7R* attenuates ASFV replication in primary macrophages and reduces virulence in vivo, demonstrating its essential role in viral pathogenesis [101,109,110]. The ability of *MGF505-7R* to manipulate both antiviral and inflammatory responses highlights its multifunctional role in immune evasion and efficient viral propagation.

Li et al. identified that ASFV *MGF505-7R* antagonizes IFN-γ-induced signaling by promoting the degradation of the host kinases JAK1 and JAK2 through distinct ubiquitination mechanisms involving the E3 ubiquitin ligase RNF125 [50]. *MGF505-7R* directly interacts with both JAK1 and JAK2, but not with STAT1. Expression of *MGF505-7R* markedly reduced JAK1 and JAK2 protein levels. Inhibition studies revealed that JAK1 degradation was blocked by the proteasome inhibitor MG132, while JAK2 degradation was prevented by both proteasomal and lysosomal inhibitors, suggesting that *MGF505-7R* triggers multiple degradation pathways. Further co-immunoprecipitation analyses identified RNF125, an E3 ubiquitin ligase, as a critical host factor recruited by *MGF505-7R* to mediate JAK1 ubiquitination [111]. *MGF505-7R* upregulates RNF125 expression in a dose-dependent manner, leading to enhanced K48-linked polyubiquitination of JAK1 and its proteasomal degradation. In parallel, *MGF505-7R* interacted with Hes5, a transcriptional regulator known to stabilize JAK2. This interaction led to the suppression of Hes5 expression, thereby destabilizing JAK2. Mechanistic findings of this study show that ASFV *MGF505-7R* employs a dual strategy: it recruits RNF125 to ubiquitinate and degrade JAK1 via the proteasome and suppresses Hes5 to destabilize JAK2. Through these actions, the JAK-STAT1 signaling pathway is effectively disrupted. Moreover, *MGF505-7R*-deficient ASFV induces higher IRF1 expression and shows reduced replication in both primary porcine alveolar macrophages and pigs. Additionally, the virulence of ASFV-Δ*MGF-505-7R* was significantly attenuated in pigs (100% survival rate), correlating with increased serum CXCL9 and IFN-γ levels [50].

### 4.10. ASFV S273R Binds with E3 Ubiquitin Ligase DCST1

The *S273R* gene encodes a 273-amino-acid protease with a molecular weight of approximately 31 kDa and is expressed during the late phase of ASFV infection. The protein is a virus-encoded cysteine protease belonging to the SUMO-1-specific protease (ULP1) family and is essential for the processing of viral polyproteins pp220 and pp62 into structural proteins required for virion assembly [112,113]. In infected cells, *S273R* localizes within viral factories, where it contributes directly to the morphogenesis of infectious particles [114]. *S273R* is highly conserved among diverse ASFV isolates, underscoring its indispensable role in the viral life cycle. Functional mutagenesis has demonstrated that disruption of *S273R* activity prevents proper cleavage of ASFV polyproteins (p62 and p220), resulting in defective core shell formation [115,116]. Beyond its role in viral maturation, *S273R* has also been implicated in the modulation of host immune responses. It has been reported to inhibit activation of the NF-κB pathway and to interfere with inflammasome activation by targeting components required for caspase-1 activation and IL-1β release [117]. *S273R* has also been shown to cleave gasdermin D in a noncanonical manner, thereby preventing pyroptosis [118].

ASFV *S273R* is a key viral protein that suppresses type I IFN signaling by selectively targeting STAT2 for degradation via K48-linked ubiquitination by the E3 ligase DCST1 (also known as Dendritic Cell-Specific Transmembrane Protein 1, DC-STAMP domain-containing protein 1) [112]. DCST1 was identified in a screen of the human ubiquitome for regulators of IFN-I signaling [119]. Overexpression of DCST1 suppresses ISRE reporter activity in response to IFN-β, whereas its silencing boosts ISRE activation and ISG expression [119,120]. Li et al. demonstrate that *S273R* specifically reduced STAT2 phosphorylation and stability. *S273R* directly binds STAT2 and enhances K48-linked polyubiquitination of STAT2. To identify the responsible E3 ubiquitin ligase, the authors screened known STAT2 regulators and found that only DCST1, but not PDLIM2 or FBXW7, interacts with *S273R*. *S273R* strengthened the association between DCST1 and STAT2, resulting in increased STAT2 ubiquitination and degradation. Moreover, knockdown of DCST1 restored STAT2 levels during ASFV infection and limited viral replication. Mapping experiments further demonstrated that DCST1 catalyzes ubiquitination at lysine 55 (K55) within the STAT2 N-terminal domain. Interestingly, this immune evasion strategy operates independently of the cysteine protease activity of *S273R*, indicating that its primary role in this context is to scaffold the interaction between STAT2 and DCST1 rather than directly cleaving substrates [51].

## 5. ASFV Proteins Disrupt NF-κB Signaling by Recruiting Host E3 Ubiquitin Ligases

**Figure 2:**
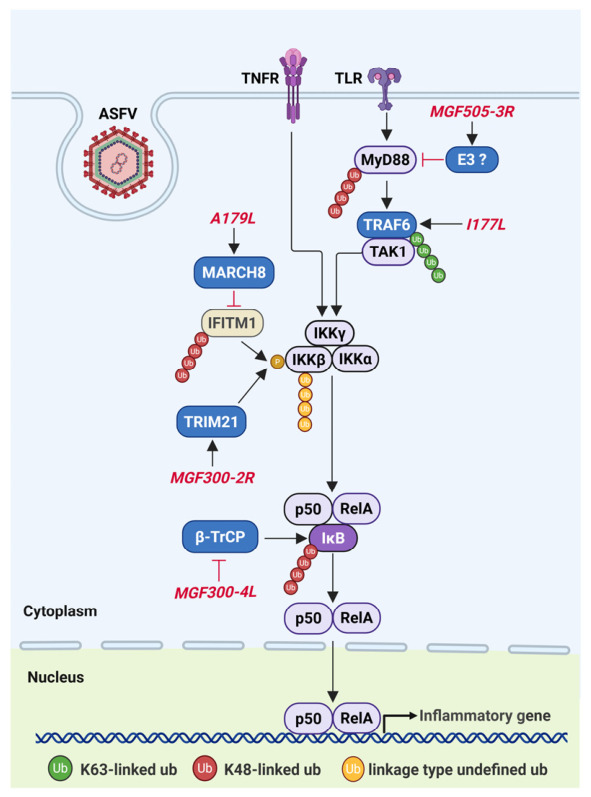
Overview of ASFV proteins that hijack or interfere with host E3 ubiquitin ligases and manipulate NF-κB signaling pathway during virus infection. The diagram summarizes the NF-κB signaling pathway targeted by ASFV proteins through direct modulation of ubiquitin-dependent signaling. Viral proteins with validated or predicted interactions are shown in red. Host factors, including E3 ligases, adaptor proteins, and signaling intermediates, are indicated at their respective subcellular locations. Ubiquitin linkages are color-coded as K63-linked (green), K48-linked (red), or undefined (yellow). (E3 ?: E3 ligase has not been defined, ub: Ubiquitination).

NF-κB signaling initiated by different pattern recognition receptors (PRRs) plays an important role in host defense against invading viruses. To initiate signaling, PRRs such as Toll-like receptors (TLRs) and tumor necrosis factor receptor (TNFR) detect extracellular or intracellular danger signals and activate downstream adaptor molecules [121]. Phosphorylation-mediated activation of the IKK complex, which consists of IKKα, IKKβ, and the regulatory subunit IKKγ (NEMO), subsequently phosphorylates the inhibitor of κB (IκB) [122], marking it for K48-linked ubiquitination and proteasomal degradation. The degradation of IκB releases the NF-κB heterodimer, primarily composed of p50 and RelA (p65), allowing its translocation from the cytoplasm into the nucleus. Once in the nucleus, NF-κB binds to specific κB elements within DNA to initiate the transcription of genes involved in inflammation, immune regulation, and cell survival [123,124]. Viruses, including ASFV, employ multiple strategies to evade NF-κB-mediated innate immune responses, thereby enhancing viral replication [125,126]. In the following section, we describe the immunomodulatory mechanisms by which ASFV proteins interfere with NF-κB signaling by recruiting host E3 ubiquitin ligases (Figure 2, Table 1 and Table 2).

### 5.1. ASFV MGF505-3R Degrades MyD88

The *MGF505-3R* gene encodes a non-structural protein in the multigene family 505, with a molecular weight of approximately 32.6 kDa. It is synthesized during the early phase of ASFV infection [127]. Although MGF505 genes are located in variable genomic regions, genes like *MGF505-3R* exhibit relatively low genetic diversity and a slow evolutionary rate. Subcellular localization studies indicate that the protein is predominantly found in the cytoplasm [76,128]. By acting as a negative regulator of the host’s innate immune response, *MGF505-3R* restricts the cGAS-STING and TBK1-IRF3 signaling pathways, thereby limiting the production of IFN-β and proinflammatory cytokines [127,129]. *MGF505-3R* has also been reported to interact with host GPX4 to facilitate ferroptosis and oxidative damage, further suppressing cellular antiviral defenses [127]. Moreover, deletion of the *MGF505-3R* gene, along with other selected genes, results in reduced virulence and attenuation in domestic pigs, making it a critical target for the development of a live-attenuated vaccine [130].

A study by Liu et al. revealed that the ASFV-encoded protein *MGF 505-3R* functions as a potent inhibitor of the host’s innate immune and inflammatory responses by targeting MyD88 [52]. MyD88 is a pivotal adaptor protein in the TLR signaling pathway required for the activation of NF-κB and the subsequent production of pro-inflammatory cytokines [131]. The study demonstrated that *MGF 505-3R* directly interacts with MyD88, leading to its K48-linked polyubiquitination and subsequent proteasomal degradation. However, the authors have not identified the E3 ubiquitin ligase responsible for ubiquitinating MyD88. Liu et al. identified that the amino acid region 89–277 of *MGF 505-3R* is the essential domain responsible for mediating the interaction with MyD88 and triggering its destabilization. By depleting MyD88, *MGF 505-3R* effectively blocks p65 phosphorylation and its nuclear translocation, broadly suppressing the expression of pro-inflammatory cytokines such as TNF-α, IL-1β, and IL-6, as well as Type I (IFN-α/β) and Type III (IFN-λ) INFs. Notably, a derived peptide (pep3R-1) based on the functional domain of *MGF 505-3R* retained these immunosuppressive properties, attenuating “cytokine storms” in a mouse model of sublethal lipopolysaccharide stimulation and alleviating Dextran Sulfate Sodium-induced colitis. Collectively, these findings highlight the dual role of *MGF 505-3R* in evading both inflammatory and antiviral pathways by disrupting a central TLR adaptor, identifying it as a significant virulence factor and a potential template for developing anti-inflammatory therapeutics [52].

### 5.2. ASFV I177L Binds with E3 Ubiquitin Ligase TRAF6

The *I177L* gene of ASFV encodes a 177-amino-acid protein that has emerged as a critical virulence determinant and pro-inflammatory effector. *I177L* is highly conserved across virulent ASFV isolates, underscoring its importance in pathogenesis. Although its precise molecular roles have only recently been elucidated, multiple studies now support its function in immune modulation and disease severity [8,53]. *I177L* facilitates activation of the TRAF6-TAK1 signaling axis. It promotes assembly of the Nucleotide-binding oligomerization domain, Leucine-rich Repeat, and Pyrin domain-containing (NLRP) 3 inflammasome, leading to robust production of inflammatory cytokines. This activity positions *I177L* as a central mediator of the dysregulated host inflammatory responses observed during acute ASFV infection [53]. Deletion of *I177L* from a virulent ASFV strain results in complete attenuation of disease in swine. Pigs infected with ASFV-Δ*I177L* remain clinically normal, show minimal viremia, no detectable virus shedding, and yet mount strong virus-specific antibody responses. Importantly, these animals are fully protected against challenge with the parental virulent strain. Compared with wild-type ASFV infection, pigs infected with the *I177L*-deficient virus exhibit markedly reduced inflammatory responses in vivo, further validating *I177L* as a driver of immunopathology. The *I177L*-deleted virus (ASFV-Δ*I177L*) has shown great promise as a live attenuated vaccine candidate. Across multiple inoculation routes, it elicits robust protective immunity without evidence of clinical disease or viral transmission, achieving sterile protection in swine [132,133]. However, concerns have emerged regarding its long-term genetic stability. Repeated passages in pigs have revealed compensatory mutations in genes such as *C257L*, which correlate with partial reversion to virulence and increased viremia [8].

ASFV *I177L* has been identified as a major pro-inflammatory viral protein that activates both NF-κB and inflammasome signaling [53]. Infection of porcine macrophages with ASFV induced IL-1β and TNF-α expression, IL-1β maturation, and caspase-1 cleavage in a time and dose-dependent manner. Mechanistically, *I177L* interacts with TRAF6 and TAK1, two central mediators of canonical NF-κB activation. *I177L* enhanced TRAF6 auto-ubiquitination via K63-linked chains, a modification required for recruitment and activation of TAK1. In the presence of *I177L*, both ubiquitination and phosphorylation of TAK1 increased significantly, thereby driving downstream activation of the TAK1/IκBα/p65 axis and the transcription of inflammatory mediators. The requirement for E3 ligase activity was demonstrated using a catalytically inactive TRAF6 mutant (C70A). Although this mutant bound *I177L* and TAK1, it failed to undergo auto-ubiquitination and could not promote TAK1 activation, abolishing the ability of *I177L* to amplify NF-κB signaling. *I177L*-deficient ASFV induced milder inflammatory responses (significantly lower serum IL-1β and TNF-α) and showed reduced replication in pigs, completely abolishing the lethality of ASFV (100% survival rate) while inducing sterile immunity. Collectively, the authors defined that *I177L* exploits the intrinsic E3 ligase function of TRAF6 to stimulate K63-linked ubiquitination, thereby stabilizing TRAF6-TAK1 complex and activating downstream inflammatory cascades [53].

### 5.3. ASFV A179L Binds with E3 Ubiquitin Ligase MARCH8

The *A179L* gene of ASFV encodes a 21 kDa Bcl-2-like protein that is expressed early during infection and is highly conserved across virulent ASFV isolates. *A179L* is located within the central conserved region of the ASFV genome and functions primarily as an inhibitor of host cell apoptosis [18,134,135]. Functionally, *A179L* mimics cellular Bcl-2 proteins and interacts with pro-apoptotic members of the Bcl-2 family, including Bax, Bak, and BH3-only proteins. Through these interactions, *A179L* prevents mitochondrial outer membrane permeabilization and inhibits the activation of caspases, thereby blocking both intrinsic and extrinsic apoptotic pathways [136]. By suppressing apoptosis, *A179L* maintains the viability of infected cells, allowing efficient viral replication and the production of infectious progeny [135,137,138]. In addition to its role in inhibiting apoptosis, A179L has been implicated in modulating host immune responses. By delaying programmed cell death, *A179L* indirectly limits the release of danger signals and inflammatory cytokines, contributing to immune evasion [55]. ASFV mutants lacking *A179L* exhibit increased apoptosis in infected macrophages and reduced viral replication, demonstrating the essential role of *A179L* in maintaining cellular integrity during infection [135].

Recent work has identified ASFV *A179L* as a viral antagonist of host innate immunity that suppresses NF-κB activation downstream of IFN-induced transmembrane protein 1 (IFITM1) [55]. Expression of *A179L* in porcine macrophages significantly reduced NF-κB-dependent reporter activity following IFITM1 stimulation, indicating that *A179L* interferes with host pro-inflammatory signaling. Mechanistically, *A179L* interacts with the host E3 ubiquitin ligase MARCH8 (Membrane Associated RING-CH 8) and co-localizes in the cytoplasm. MARCH8 is a RING-CH type E3 ubiquitin ligase playing a key role in protein trafficking, immune regulation, and antiviral defense. Reports demonstrate that HIV, influenza A, and hepatitis C virus also exploit MARCH8 activity to evade host immunity by interfering with substrate recognition or redirecting ubiquitination to favor viral persistence [139,140]. Ubiquitination assays revealed that *A179L* enhances MARCH8-mediated K27 and K48-linked ubiquitination of IFITM1, targeting it for proteasome-dependent degradation. Treatment with proteasome inhibitors restored IFITM1 levels and rescued NF-κB activation. Collectively, these findings demonstrate that ASFV *A179L* suppresses NF-κB signaling by exploiting MARCH8 to ubiquitinate and degrade IFITM1 [55].

### 5.4. ASFV MGF300-2R Binds with E3 Ubiquitin Ligase TRIM21

The *MGF300-2R* gene encodes a protein of roughly 280–300 aa, with an estimated molecular weight of ~33 kDa, and is expressed during the early stages of ASFV infection. Like other members of the MGF300, *MGF300-2R* has been linked to viral-host interaction, modulation of immune responses, and maintenance of pathogenicity [141,142]. *MGF300-2R* is highly conserved among virulent field isolates but is often disrupted or absent in naturally attenuated strains [56,141]. Within infected macrophages, *MGF300-2R* is predominantly cytoplasmic and associates with viral replication compartments, consistent with a role in coordinating virus–host interactions. Experimental deletion of *MGF300-2R* has been shown to impair ASFV replication in primary porcine macrophages and to attenuate disease in swine models [143]. In addition, accumulating evidence identifies *MGF300-2R* as an innate immune antagonist that suppresses host antiviral signaling, thereby enabling ASFV to establish productive infection [54,141].

Recent work by Lu et al. identified ASFV *MGF300-2R* as a viral protein that targets the NF-κB signaling pathway through the host E3 ubiquitin ligase TRIM21 [54]. Mass spectrometry analyses of *MGF300-2R* interacting proteins in HEK293T cells revealed multiple candidate E3 ligases, including HUWE1, ARIH2, PRPF19, UBR5, OBI1, and TRIM21. *MGF300-2R* binds and co-localizes with TRIM21 in the cytoplasm, positioning it to manipulate host signaling complexes. Mechanistically, TRIM21 functions as a catalyst in the transfer of ubiquitin from E2-conjugating enzymes to IKKβ. Co-expression of TRIM21 with IKKβ induced dose-dependent ubiquitination and subsequent proteasomal degradation of IKKβ. Importantly, deletion of TRIM21 in HEK293T cells abolished *MGF300-2R*-mediated IKKβ degradation, establishing that TRIM21 is indispensable for this process. The loss of IKKβ impairs phosphorylation of IκBα, thereby preventing its degradation and retaining NF-κB in the cytoplasm. Porcine infection models demonstrate that *MGF300-2R*-deficient ASFV shows significantly reduced replication and virulence in pigs [54].

### 5.5. ASFV MGF300-4L Binds with E3 Ubiquitin Ligase β-TrCP

The *MGF300-4L* gene encodes a protein of 300 aa, with a predicted molecular weight of ~34 kDa, and is expressed during the early phase of ASFV infection. *MGF300-4L* is well conserved across virulent ASFV isolates but may display variability in attenuated strains, consistent with its role in supporting viral fitness and pathogenicity [144]. Within infected cells, *MGF300-4L* localizes predominantly in the cytoplasm, with enrichment near viral replication sites [56,144]. Functional studies have demonstrated that deletion of *MGF300-4L* attenuates ASFV replication in primary macrophages and reduces viral virulence in swine, underscoring its contribution to efficient viral propagation and disease outcome [56].

The study by Wang et al. characterized ASFV *MGF300-4L* as a potent viral antagonist of NF-κB signaling through the interaction with the E3 ubiquitin ligase β-TrCP (also known as β-transducin repeat containing protein or F-Box and WD repeats protein beta-1A, FBXW1) [56]. β-TrCP functions as a substrate recognition subunit of the SCF (Skp1-Cullin1-F-box) E3 ubiquitin ligase complex and plays essential roles in immune regulation, cell cycle progression, and oncogenesis [145,146]. Several viral proteins, including HIV-1 Vpu and poxvirus proteins, directly hijack or block β-TrCP to evade NF-κB-mediated antiviral responses, reflecting its centrality as a restriction point in host–pathogen interactions [147,148]. Mechanistically, *MGF300-4L* suppresses NF-κB activation through a dual strategy targeting key regulatory nodes of the pathway. First, *MGF300-4L* directly interacts with IKKβ and promotes its selective degradation via the lysosomal pathway; this is consistent with lysosomal inhibitors (BafA1, 3-MA) rescuing IKKβ stability, whereas inhibition of the proteasome pathway (Lac, MG132) had minimal effect. Second, *MGF300-4L* stabilizes IκBα by interfering with its ubiquitination and degradation. Under normal conditions, IκBα is phosphorylated on serine residues (S32, S36) within the DSGX2-3S motif, enabling recognition by β-TrCP. β-TrCP-mediated K48-linked polyubiquitination targets IκBα for proteasomal degradation, releasing NF-κB for nuclear translocation and transcriptional activation [148]. *MGF300-4L* competes with IκBα for β-TrCP binding, thereby inhibiting IκBα ubiquitination and proteasomal degradation. Consequently, NF-κB remains sequestered in the cytoplasm, preventing the transcription of IL-1β and TNF-α. *MGF300-4L*-deficient ASFV shows reduced replication in macrophages and pigs, leading to higher serum levels of IL-1β and TNF-α, and attenuated virulence and pathogenicity in pigs (50% survival rate) [56].

## 6. ASFV Proteins Disrupt Apoptotic Signaling by Recruiting Host E3 Ubiquitin Ligases

### ASFV MGF360-9L Binds with E3 Ubiquitin Ligase RNF114

The *MGF360-9L* gene encodes a small protein expressed during the early phase of ASFV infection. *MGF360-9L* is conserved among many virulent ASFV isolates, although it is often absent or truncated in naturally attenuated strains, highlighting its role in pathogenicity [110,149,150]. In infected cells, *MGF360-9L* localizes to the cytoplasm and viral factories, where it exerts immunomodulatory functions rather than direct roles in virion assembly. Functional studies have demonstrated that *MGF360-9L* inhibits type I IFN responses by targeting JAK/STAT signaling downstream of the IFN receptor [142,151]. This inhibition blocks ISG transcription, allowing ASFV to evade the host’s first line of defense. Deletion of *MGF360-9L*, alone or in combination with other *MGF110/505* genes, has been shown to attenuate the virus in pigs and enhance host IFN production, supporting its contribution to immune suppression and viral persistence [110,149]. Together, these findings place *MGF360-9L* within a class of ASFV immunomodulators that fine-tune host virus interactions.

The study by Yang et al. identified ASFV *MGF360-9L* as a viral protein that subverts host defenses through targeted degradation of the anti-apoptotic protein HAX1 via the E3 ubiquitin ligase RNF114 [57]. RNF114 (also known as Ring Finger Protein 114 or Zinc Finger Protein 313, Zinc Finger Protein 228) is a RING-type E3 ubiquitin ligase initially identified as a psoriasis susceptibility gene in genome-wide association studies [152]. Reports demonstrate that RNF114 restricts porcine reproductive and respiratory syndrome virus (PRRSV) replication by targeting the viral nonstructural protein 12 (Nsp12) for proteasome-dependent degradation via K27-linked polyubiquitination [153]. In the study by Yang et al., based on published *MGF360-9L* and host protein interaction networks, HAX1 was identified as a potential binding protein for *MGF360-9L*. *MGF360-9L* directly binds to HAX1, with multiple regions of the viral protein (1–161 aa, 106–235 aa, and 209–350 aa) mediating this interaction, while the critical binding domain on HAX1 was localized to its C-terminal region (160–279 aa). Overexpression of HAX1 significantly inhibited ASFV replication, whereas *MGF360-9L* promoted HAX1 degradation. Inhibition studies demonstrated that this degradation is proteasome-dependent, as treatment with MG132, a proteasome inhibitor, blocked HAX1 turnover. RNF114 interacted with both *MGF360-9L* and HAX1, and co-expression experiments confirmed a three-way complex formation. Ubiquitination assays showed that RNF114 catalyzed K48-linked polyubiquitination of HAX1. Importantly, *MGF360-9L* enhanced the association between RNF114 and HAX1, thereby accelerating K48-linked ubiquitination and HAX1 degradation. Collectively, these findings of this study demonstrate that *MGF360-9L* acts as a viral effector that hijacks the host E3 ligase RNF114 to facilitate K48-linked ubiquitination and degradation of HAX1, thereby enhancing virus replication [57].

## 7. Conclusions and Future Directions

Across the studies discussed, several recurring patterns emerge, highlighting a coordinated strategy by ASFV to exploit host E3 ubiquitin ligases. A central theme is the preferential targeting of key innate immune signaling nodes, particularly the cGAS-STING axis, RIG-I-MAVS axis, TLRs, and the TNFR-mediated NF-κB pathways, which serve as critical checkpoints for antiviral responses. Multiple ASFV proteins converge on these pathways, either by inhibiting upstream sensors such as RIG-I or by disrupting downstream signaling components including TBK1, IRF3, and IKKβ. Mechanistically, interference with K63-linked ubiquitination appears to be a dominant strategy. ASFV proteins frequently suppress K63-linked ubiquitination to block activation of immune signaling complexes, while in parallel promoting K48-linked ubiquitination to drive proteasomal degradation of key host factors such as IRF3, STAT2, JAK1, and IKKβ. This dual approach allows the virus to both prevent signal initiation and actively remove critical signaling molecules.

Notably, different viral proteins often target the same host molecules or pathways through distinct mechanisms. Among the host E3 ubiquitin ligases discussed in this review, TRIM21 and TRAF6 emerge as particularly attractive candidates that are repeatedly targeted by ASFV proteins. TRAF6 is manipulated by both *A151R* and *I177L* through distinct mechanisms. *A151R* suppresses TRAF6-mediated activation of TBK1 and type I IFN signaling, whereas *I177L* exploits TRAF6-dependent ubiquitination to promote NF-κB activation and inflammasome signaling. TRIM21 is targeted by three ASFV proteins, including *CP204L*, *MGF360-14L*, and *MGF300-2R*. *CP204L* inhibits TRIM21-mediated K27-linked ubiquitination of MAVS, thereby suppressing antiviral signaling, whereas *MGF360-14L* recruits TRIM21 to promote K48-linked ubiquitination and degradation of IRF3. In addition, *MGF300-2R* utilizes TRIM21 to facilitate IKKβ degradation, thereby suppressing NF-κB activation. The recurrent targeting of TRIM21 and TRAF6 by multiple ASFV proteins highlights their critical roles in coordinating antiviral innate immune responses. It suggests that they are major host-dependency factors exploited during infection. Whether ASFV similarly exploits host E3 ubiquitin ligases in its natural hosts (warthogs) and arthropod vectors (soft ticks) remains unknown. Investigating ubiquitination pathways in these natural reservoirs may provide valuable insights into ASFV host adaptation, persistence, and transmission throughout its natural infection cycle. ASFV is not unique in its ability to manipulate host E3 ubiquitin ligases. Similar strategies have been described in other large DNA viruses, including herpesviruses and poxviruses, which exploit ubiquitin-dependent pathways to suppress innate immune responses and promote viral replication. For example, several herpesviruses and poxviruses target key components of the cGAS-STING, RIG-I-MAVS, and NF-κB pathways through ubiquitin-mediated degradation or signaling interference [148,154,155,156]. However, unlike many herpesviruses and poxviruses that encode viral ubiquitin ligases or ubiquitin-like proteins [157,158,159,160] ASFV appears to rely predominantly on the recruitment and reprogramming of host E3 ubiquitin ligases. This extensive dependence on the host ubiquitination machinery suggests that future studies aimed at preserving or restoring the activity of these E3 ligases may yield promising host-directed antiviral strategies that are less vulnerable to viral resistance than conventional virus-targeted therapeutics.

The attenuation observed following deletion of several ASFV genes that manipulate host E3 ubiquitin ligases highlights the critical role of ubiquitin-dependent immune evasion in ASFV pathogenesis. Several promising candidates, including ASFV-Δ*I177L*, ASFV-Δ*A151R*, and strains lacking MGF360/MGF505 family genes, specifically subvert host immunity through ubiquitin-dependent protein degradation or by interference with signaling. For example, the deletion of *MGF-505-7R* significantly attenuates the virus in swine, resulting in a 100% survival rate and increased serum IFN-γ levels. Similarly, ASFV-Δ*MGF-360-10L* exhibits compromised replication in primary macrophages and complete attenuation in porcine models. ASFV-Δ*I267L* induces significantly higher levels of serum IFN-β and results in an 83% survival rate in pigs. Furthermore, ASFV-Δ*I177L* not only abolishes lethality but also induces sterile immunity. However, the redundancy of ASFV immune evasion mechanisms remains a significant hurdle. The deletion of a single gene may not entirely abolish immune suppression because the virus can target the same pathway through multiple complementary proteins. Given concerns regarding genetic stability and potential reversion to virulence, a deeper mechanistic understanding of ASFV host E3 ligase interactions is essential. This knowledge will facilitate the rational design of next-generation live-attenuated vaccines that incorporate multiple stable deletions, ensuring both a superior safety profile and enhanced immunogenicity.

Immune evasion strategies allow ASFV to establish persistent infection, undermine host defenses, and contribute to its high pathogenicity. Despite substantial research progress in recent years, our understanding of the interplay between ASFV and ubiquitin remains incomplete. Critical questions persist regarding the structural basis of viral protein-E3 interactions, the precise ubiquitin chain linkages manipulated during infection, and how these modifications reshape cellular pathways beyond innate immunity, including autophagy, apoptosis, and inflammatory signaling. Also, most studies have focused on individual virus–host interactions, whereas the global landscape of ubiquitin-dependent signaling during ASFV infection remains largely unexplored. Therefore, future research should prioritize comprehensive proteomic, ubiquitinomic, and structural studies to systematically map ASFV–host ubiquitin interactions and define the molecular basis of viral protein and host E3 ligase engagement. In particular, proximity-labeling proteomic approaches, such as TurboID or APEX2, may facilitate the identification of transient and weak interactions between ASFV proteins and host ubiquitination machinery. At the same time, quantitative ubiquitinomics coupled with mass spectrometry could reveal global changes in the architecture of ubiquitin linkages during infection. Functional genomic approaches, including genome-wide or targeted CRISPR-Cas9 screens and siRNA-based knockdown studies, will be valuable for identifying host E3 ligases, de-ubiquitinases, and associated factors that are essential for viral replication and immune evasion. Such studies will not only improve our understanding of ASFV pathogenesis but may also reveal novel host-directed therapeutic targets that are less susceptible to viral escape mutations. Integrating these data with in vivo infection models will be essential to determine how ubiquitin manipulation contributes to virulence and host adaptation. Importantly, exploring host-directed interventions such as targeting specific E3 ligases, ubiquitin chain regulators, or de-ubiquitinases could open new avenues for antiviral strategies. However, host E3 ligases regulate diverse physiological processes; systemic targeting may lead to unintended disruption of normal cellular homeostasis and immune function. Therefore, therapeutic strategies targeting host E3 ligases should be approached with caution, prioritizing tissue-specific delivery or selective transient modulation to maximize antiviral efficacy while minimizing off-target toxicity. Continued investigation of ASFV exploitation of the ubiquitin system will not only advance our understanding of viral immune evasion but also facilitate the rational development of next-generation vaccines and host-directed therapeutics. Addressing these gaps is critical for establishing sustainable control measures against this devastating pathogen and enhancing global preparedness against future ASFV incursions.

## Figures and Tables

**Table 1:** ASFV proteins that target host E3 ubiquitin ligases and their mechanisms of action.

ASFV Gene	E3 Ligase	Host Target Protein	Type of Ubiquitination	Mechanism	Ref.
*I267L*	Riplet	RIG-I	K63	*I267* binds Riplet and competitively disrupts the Riplet–RIG-I interaction, blocking K63-linked polyubiquitination of RIG-I.	[42]
*E248R*	TRIM25	RIG-I	K63	*E248R* binds the coiled-coil domain of TRIM25, blocking its multimerization and abolishing TRIM25-mediated K63-linked ubiquitination of RIG-I.	[43]
*MGF505-9R*	RNF125	RIG-I		*MGF505-9R* binds with RNF125 and induces autoubiquitination and degradation, leading to the subversion of RNF125-mediated K48 ubiquitination of signaling molecules	[44]
*CP204L*	TRIM21	MAVS	K27	*CP204L* binds TRIM21 and MAVS, competitively blocking TRIM21-mediated K27-linked ubiquitination of MAVS.	[45]
*A151R*	TRAF6	TBK1	K63	*A151R* binds TRAF6 and promotes its apoptosis-mediated degradation, disrupting the TRAF6-TBK1 interaction and abolishing TRAF6-mediated K63-linked ubiquitination of TBK1.	[46]
*I215L*	RNF138	RNF128	Undefined	*I215L* binds RNF138, enhancing its interaction with RNF128 and promoting RNF138-mediated degradation of RNF128, thereby reducing K63-linked polyubiquitination of TBK1.	[47]
*MGF360-14L*	TRIM21	IRF3	K63	*MGF360-14L* recruits TRIM21 to mediate K63-linked ubiquitination and proteasomal degradation of IRF3.	[48]
*MGF360-10L*	HERC5	JAK1	K48	*MGF360-10L* recruits HERC5 to mediate K48-linked ubiquitination of JAK1 to promote its proteasomal degradation	[49]
*MGF505-7R*	RNF125	JAK1	Undefined	*MGF505-7R* upregulates RNF125 expression, promoting JAK1 ubiquitination and degradation.	[50]
*S273R*	DCST1	STAT2	K48	*S273R* recruits E3 ligase DCST1 to STAT2, promoting K48-linked polyubiquitination and proteasomal degradation of STAT2.	[51]
*MGF505-3R*	Unknown	MyD88	K48	*MGF505-3R* promotes ubiquitin-mediated proteasomal degradation of MyD88.	[52]
*I177L*	TRAF6	TRAF6	K63	*I177L* promotes TRAF6 auto-ubiquitination, enhancing TRAF6-TAK1 interaction and TAK1 activation	[53]
*MGF300-2R*	TRIM21	IKKβ	Undefined	*MGF300-2R* recruits TRIM21 to mediate ubiquitination and degradation of IKKβ.	[54]
*A179L*	MARCH8	IFITM1	K48	*A179L* recruits MARCH8 to IFITM1, promoting K48-linked ubiquitination and proteasomal degradation of IFITM1.	[55]
*MGF300-4L*	β-TrCP	IκBα	K48	*MGF300-4L* competitively blocks β-TrCP binding to IκBα, preventing its ubiquitination-dependent degradation.	[56]
*MGF360-9L*	RNF114	HAX1	K48	*MGF360-9L* recruits RNF114 to HAX1, facilitating K48-linked ubiquitination and proteasomal degradation of HAX1.	[57]

K27: K27-linked ubiquitination, K48: K48-linked ubiquitination, K63: K63-linked ubiquitination, Undefined: Specific linkage type has not been defined in the present study.

**Table 2:** ASFV proteins that exploit host ubiquitin machinery for immune evasion: affected signaling pathways and phenotypes of gene-deleted viruses (in vitro and in vivo phenotypes mentioned in the table are only based on the referred article).

ASFV Gene	Expression Phase	Signaling Pathway Affected	In Vitro Deletion Phenotype	In Vivo Deletion Phenotype	Ref.
*I267L*	Early stage	Type I IFN signaling	Impaired viral replication	Attenuated virulence in pigs	[42]
*E248R*	Middle-late stage	Type I IFN signaling	Enhances IFN-β production and inhibits ASFV replication	Not reported	[43]
*MGF505-9R*	Early stage	Type I IFN signaling	Reduce IFN-β and IL-1β, IL-10 production	Not reported	[44]
*CP204L*	Early stage	Type I IFN signaling	Reduces ASFV replication	Not reported	[45]
*A151R*	Early stage	Type I IFN signaling	Inhibits ASFV replication and enhances IFN-β	Not reported	[46]
*I215L*	Early stage	Type I IFN signaling	Enhances IFN-β promoter and inhibits ASFV replication	Not reported	[47]
*MGF360-14L*	Early stage	Type I IFN signaling	Not reported	Not reported	[48]
*MGF360-10L*	Early stage	JAK–STAT signaling	Inhibits ASFV replication; upregulated ISG15 and ISG56 induction	Attenuated virulence in pigs	[49]
*MGF505-7R*	Early stage	JAK–STAT signaling	Inhibits ASFV replication, enhances IRF1 expression	Attenuated virulence in pigs	[50]
*S273R*	Late stage	JAK–STAT signaling	Not reported	Not reported	[51]
*MGF505-3R*	Early stage	Type I, III IFN and NF-κB	Not reported	Not reported	[52]
*I177L*	Late stage	NF-κB and NLRP3 inflammasome	Inhibits ASFV replication, reduce NF-κB/NLRP3 signaling	Attenuated virulence in pigs, reduces IL-1β and TNF-α	[53]
*MGF300-2R*	Early stage	NF-κB signaling	Not reported	Not reported	[54]
*A179L*	Early to late stage	NF-κB signaling	Enhances NF-κB activation and pro-inflammatory cytokines	Not reported	[55]
*MGF300-4L*	Early stage	NF-κB signaling	Reduced ASFV replication and increased IL-1β and TNF-α production	Attenuated virulence in pigs, reduces IL-1β and TNF-α	[56]
*MGF360-9L*	Early stage	Apoptosis signaling	Not reported	Not reported	[57]

## Data Availability

No new data were created or analyzed in this study. Data sharing is not applicable to this article.
